# IMPACT OF AN INTERDISCIPLINARY CLASS ON FIRST-YEAR UNDERGRADUATE STUDENT KNOWLEDGE AND ATTITUDES TOWARD AGING

**DOI:** 10.1093/geroni/igad104.2115

**Published:** 2023-12-21

**Authors:** Matthew Picchiello, Dylan Agran, Tim Chung, Brian Carpenter, Susy Stark, Nancy Morrow-Howell

**Affiliations:** Washington University in St. Louis, St. Louis, Missouri, United States; Washington University in St. Louis, St. Louis, Missouri, United States; Washington University in St. Louis, St. Louis, Missouri, United States; Washington University in St. Louis, St. Louis, Missouri, United States; Washington University in St. Louis, St. Louis, Missouri, United States; Washington University in St. Louis, St. Louis, Missouri, United States

## Abstract

Undergraduate classes on aging have the potential to challenge ageist beliefs and alter students’ academic and career trajectories. For the past eight years, we have led an introductory, interdisciplinary class on aging, bringing first-year students and older adults together to discuss various facets of aging and share their perceptions and experiences. Both prior to and after the class, students in the class (n = 494), and a group of their age-matched peers not enrolled in the class (n = 620), completed an online survey measuring attitudes and knowledge about aging and older adults. Repeated measure analyses revealed significant group-by-time interactions across several measures. While students who were not in the class did not show statistically significant changes across measures, students in the class displayed statistically significant increases in knowledge about aging, F(1, 760) = 108.133, ηp2 = 0.125; decreases in aging related anxiety F(1, 766) = 51.947, ηp2 = 0.064; and more favorable expectations about their own aging, F(1, 627) = 45.598, ηp2 = 0.068. Moreover, students in the class endorsed fewer ageist beliefs over time, F(1,768) = 135.313, ηp2 = 0.15, all p’s < 0.05. Despite these promising changes, overall intent to engage in an aging-related career did not statistically change across groups, F(1, 113) = 2.948, p > 0.05. Results suggest that exposing students to information about aging in an intergenerational classroom has the potential to mitigate biases against older adults, though challenges remain in maintaining student interest in aging-related careers.

